# Association between arsenic, cadmium, manganese, and lead levels in private wells and birth defects prevalence in North Carolina: a semi-ecologic study

**DOI:** 10.1186/1471-2458-14-955

**Published:** 2014-09-15

**Authors:** Alison P Sanders, Tania A Desrosiers, Joshua L Warren, Amy H Herring, Dianne Enright, Andrew F Olshan, Robert E Meyer, Rebecca C Fry

**Affiliations:** Department of Environmental Sciences and Engineering, Gillings School of Global Public Health, University of North Carolina, Chapel Hill, NC USA; Department of Epidemiology, Gillings School of Global Public Health, University of North Carolina, Chapel Hill, NC USA; Department of Biostatistics, Gillings School of Global Public Health, University of North Carolina, Chapel Hill, NC USA; Division of Public Health, Health and Spatial Analysis Branch, State Center for Health Statistics, Raleigh, NC USA; Department of Maternal and Child Health, Gillings School of Global Public Health, University of North Carolina, Chapel Hill, NC USA; Division of Public Health, North Carolina Birth Defects Monitoring Program, State Center for Health Statistics, Raleigh, NC USA; Department of Preventive Medicine, Icahn School of Medicine at Mount Sinai, New York, NY USA; Department of Biostatistics, Yale School of Public Health, Yale University, New Haven, CT USA

**Keywords:** Metals, Arsenic, Cadmium, Manganese, Lead, Congenital heart defect, Drinking water, Birth defects, Congenital anomalies

## Abstract

**Background:**

Toxic metals including arsenic, cadmium, manganese, and lead are known human developmental toxicants that are able to cross the placental barrier from mother to fetus. In this population-based study, we assess the association between metal concentrations in private well water and birth defect prevalence in North Carolina.

**Methods:**

A semi-ecologic study was conducted including 20,151 infants born between 2003 and 2008 with selected birth defects (cases) identified by the North Carolina Birth Defects Monitoring Program, and 668,381 non-malformed infants (controls). Maternal residences at delivery and over 10,000 well locations measured for metals by the North Carolina Division of Public Health were geocoded. The average level of each metal was calculated among wells sampled within North Carolina census tracts. Individual exposure was assigned as the average metal level of the census tract that contained the geocoded maternal residence. Prevalence ratios (PR) with 95% confidence intervals (CI) were calculated to estimate the association between the prevalence of birth defects in the highest category (≥90^th^ percentile) of average census tract metal levels and compared to the lowest category (≤50^th^ percentile).

**Results:**

Statewide, private well metal levels exceeded the EPA Maximum Contaminant Level (MCL) or secondary MCL for arsenic, cadmium, manganese, and lead in 2.4, 0.1, 20.5, and 3.1 percent of wells tested. Elevated manganese levels were statistically significantly associated with a higher prevalence of conotruncal heart defects (PR: 1.6 95% CI: 1.1-2.5).

**Conclusions:**

These findings suggest an ecologic association between higher manganese concentrations in drinking water and the prevalence of conotruncal heart defects.

**Electronic supplementary material:**

The online version of this article (doi:10.1186/1471-2458-14-955) contains supplementary material, which is available to authorized users.

## Background

The toxic metals arsenic, cadmium, manganese, and lead are known developmental toxicants in humans [1–4]. These metals are able to cross the placental barrier resulting in *in utero* exposures to the developing fetus [5–7]. Some of the detrimental health outcomes associated with exposure to these metals include spontaneous abortion, stillbirth, low birth weight, preterm birth, reduced fetal growth, impaired neurodevelopment, and congenital malformation [8–15].

Birth defects are a leading cause of infant mortality in the U.S. [16, 17], yet 60-70% have no known cause [18]. In epidemiologic studies, toxic metals including arsenic, cadmium, and lead have been associated with congenital structural malformations (reviewed in [9]). Exposure to arsenic- and lead-contaminated drinking water has been associated with an increased occurrence of congenital heart defects (CHDs) [19–21] as well as neural tube defects (NTDs) in human populations [22]. While these studies demonstrated elevated associations, some of the reported 95% confidence intervals were consistent with unity. In contrast, the teratogenicity of arsenic, cadmium, manganese, and lead has been demonstrated in animal models, specifically affecting cardiac, limb, musculoskeletal, craniofacial, and central nervous system development [10, 23–26]. It is clear from animal studies that metal exposure can result in structural malformation; however, there have been few population-based human studies that have examined such relationships.

Diet and drinking water are common sources of metal exposure in non-occupationally exposed individuals. In North Carolina, 2.3 million residents use private wells for drinking water [27], and the water quality of these wells is not federally regulated [28]. Notably, several statewide assessments of North Carolina private wells have indicated geographical regions with naturally-occurring manganese [29] and arsenic [30–32]. Arsenic levels in some wells can reach up to ~800 ppb a level that is 80 times the current EPA Maximum Contaminant Level (MCL) standard (10 ppb) in public distributions systems and clearly associated with disease risk [30]. These data support that ingestion of metal-contaminated drinking water may be a public health concern in North Carolina. No previous studies in North Carolina have been conducted to investigate the potential association between birth defects and metal concentrations in private well water.

In the present study we examined private well water levels of arsenic, cadmium, manganese, and lead across North Carolina, and used a semi-ecologic study design to estimate the association between metal levels and specific birth defect phenotypes. As individual level data on maternal water consumption were not available, maternal drinking water metal levels were assigned based on geocoded residence within a census tract. The prevalence of twelve specific defect types or groups of defects was compared between census tracts with high versus low average metal levels. This work represents the first effort to assess the association between statewide levels of metals in North Carolina wells with birth defects.

## Methods

### Study design and study population

This study was reviewed by the UNC IRB (#11-0414) and was approved according to regulatory category 5 under the National Institutes of Health description on Research on Human Specimens (44 CFR 46.101(b)). A statewide semi-ecologic study was conducted from a baseline study population that included livebirths between January 2003 and December 2008 that comprised 24,704 infants with birth defects (cases) and 725,690 non-malformed controls in North Carolina. Infants from non-singleton births (n = 25,069), without a geocoded residence at delivery (n = 38,206), or case infants with known chromosomal abnormalities (n = 1,472) were considered ineligible and excluded from this study.

Individual-level data regarding maternal source of drinking water and frequency of consumption were not available. Thus, the census tract corresponding to maternal residence at delivery served as the unit of analysis in this semi-ecologic study. The analysis included 1,563 census tracts comprising 5,271 block groups across North Carolina. Private well water metal data were provided by the North Carolina Department of Health and Human Services (DHHS) within the Division of Public Health. The well water database contained metal concentrations collected from private wells across the state between 1998 and 2010 measured by inductively coupled plasma-mass spectrometry. This database contains historical records of well water samples collected by and analyzed at the State Laboratory of Public Health in Raleigh, North Carolina as we have previously summarized [30]. State or federal laws do not require the testing of existing private wells in North Carolina, though state law requires testing of new wells since 2008. As such, the data in this study represent metal analyses from newly constructed private wells since July 2008 (according to North Carolina rule 15A NCAC 18A.3802) and wells tested between 1998 and 2010 where owners requested testing of existing wells. The total number of wells tested was between 22,000 and 76,000 wells depending on the metal that was analyzed.

### Outcome classification

Case infants with selected birth defects were identified by the North Carolina Birth Defects Monitoring Program (BDMP). The BDMP is a statewide active surveillance program covering all 100 North Carolina counties [33]. Cases were livebirths between 2003 and 2008 identified through systematic review and abstraction of medical records by BDMP field staff. Diagnoses were confirmed by supporting documentation in the medical record, such as surgical or autopsy reports, medical imaging, and physical exams. Selected birth defects in this analysis included a spectrum of phenotypes comprising NTDs, CHDs, gastrointestinal, genitourinary, and musculoskeletal defects that are of prioritized interest by the Centers for Disease Control and Prevention. Non-malformed controls infants were identified from birth certificate records during the same years.

The following twelve structural defects or groups of defects were included in this study: (1) spina bifida without anencephaly (n = 218); (2) anotia and microtia (n = 94); (3) conotruncal heart defects including common truncus, Tetralogy of Fallot (TOF), and transposition of the great arteries (TGA) (n = 435); (4) atrioventricular septal defects (AVSD) and endocardial cushion defects (ECD) (n = 150); (5) hypoplastic left heart syndrome (HLHS) (n = 142); (6) cleft palate (CP) (n = 351); (7) cleft lip with or without CP (n = 516); (8) esophageal atresia (EA) and tracheo-esophageal fistula (TEF) (n = 140); (9) pyloric stenosis (n = 1,204); (10) reduction defects of the upper and lower limbs (n = 255); (11) gastroschisis (n = 215); and (12) hypospadias (n = 1,994).

### Exposure assessment

Environmental metals of interest included arsenic, cadmium, manganese, and lead. As described above, more than 22,000 measurements of metal concentrations in private wells were collected by the North Carolina DHHS across the state between 1998 and 2010. Only private wells with a corresponding global positioning system (GPS) coordinate or with a complete geocoded address were considered in this study (50-67% of wells sampled statewide depending on the metal analyzed) to minimize the impact of geocoding error. GPS coordinates were standardized to a decimal degree format and private well locations were geocoded according to previously described methods [30]. The number of geocoded wells varied by metal where 46,286 (67%) wells had arsenic measures, 11,381 (50%) wells had cadmium measures, 46,022 (65%) wells had manganese measures, and 46,059 (60%) wells had lead measurements. Geocoded well locations were then assigned to North Carolina census tracts that corresponded to the 2000 census using ESRI ArcGIS^TM^ 9.3 (Redlands, CA).

The average level of each of the four metals (arsenic, cadmium, manganese, and lead) was calculated within each census tract across the state. The distributions of average metal levels were mapped using ArcGIS. Census tracts with fewer than ten well measures for each contaminant over the total time period were excluded from further analysis to reduce exposure misclassification in poorly sampled tracts. Approximately 50% of census tracts were excluded due to limited sampling.

Maternal residence at delivery for cases and controls was geocoded by the North Carolina State Center for Health Statistics. Cases and controls were assigned to census tracts in North Carolina based on geocoded maternal residence at delivery using ArcGIS. A dichotomous exposure contrast was developed to compare “exposed” and “unexposed” census tracts at delivery. First, the distribution of average metal concentrations across all tracts with at least ten well water measures was examined, and the 50^th^ and 90^th^ percentiles of average concentration was calculated for each metal. Then, an exposure status was assigned to each census tract for each metal where residences were considered “exposed” if the average concentration of the metal within the corresponding tract was equal to or greater than the 90^th^ percentile of metal concentration across all tracts combined. Likewise, tracts were considered “unexposed” if the average concentration of the metal within the corresponding tract was less than or equal to the 50^th^ percentile of metal concentration across all tracts combined. Thus, the final dichotomous exposure contrast compared exposed census tracts (a tract where metal concentrations exceeded the 90^th^ percentile of the distribution of metal concentrations across the state) and unexposed census tracts. The 50^th^ and 90^th^ percentile of census tract average levels were as follows: 1.29 and 2.54 ppb for arsenic, 0.54 and 1.82 ppb for cadmium, 41.55 and 139.69 ppb for manganese, and 3.52 and 7.28 ppb for lead.

### Covariates

Maternal and infant characteristics were obtained for cases and controls from the North Carolina birth certificate. Covariates were selected *a priori* using a directed acyclical graph approach. The final set included in the statistical analysis were maternal age (continuous), race/ethnicity (categorized as non-Hispanic white, non-Hispanic Black, Hispanic, or Other), and education (categorized as mother’s highest level of completed education as less high school, high school, or greater than high school).

### Statistical analysis

Crude and adjusted estimates of the association between metal concentrations in drinking water and the prevalence of each birth defect within census tracts were calculated by log-linear regression using SAS 9.3 (SAS Institute Inc., Cary, North Carolina). Prevalence ratios (PRs) with 95% confidence intervals (CIs) were calculated to estimate the association between dichotomized metal exposure status for each metal and the prevalence of birth defects within census tracts, adjusted for maternal age at delivery, race/ethnicity and education status. Any subjects with missing data for one or more covariates were excluded from the adjusted statistical model. To account for multiple testing, Bonferroni-corrected PRs with 99.9% CI estimates (alpha = 0.001) were also calculated.

To examine potential inter-metal correlation, Spearman’s rank correlation was used to examine the relationship between each pairwise metal level averaged to the ecologic unit. Corresponding Spearman’s rank coefficients (r) and p-values were calculated.

Given the correlations observed between levels of arsenic and manganese, a multiplicative interaction effect was estimated for the association between residence in areas of both higher arsenic and manganese levels and the prevalence of each birth defect category. Specifically, the prevalence of birth defects among infants residing in census tracts where both arsenic and manganese levels were greater than or equal to the 90^th^ percentile exposed category were compared to areas where both arsenic and manganese were less than or equal to the 50^th^ percentile unexposed category. Crude and adjusted PRs and corresponding 95% CIs were calculated using log-linear regression as described above.

### Sensitivity analyses

A sensitivity analysis was conducted using the census block group as the ecologic unit of analysis to determine whether the pattern of findings observed at the tract level was robust. The analysis included 5,271 block groups across North Carolina. Although the smaller block group level as the unit of exposure resulted in a finer geographical exposure assessment, fewer study subjects (roughly 50% of the cohort included with the tract ecologic unit analysis) were included since a larger proportion of the block groups did not have adequate well sampling data (greater than 75%). Maternal residence at delivery and the private well locations were assigned to North Carolina block groups corresponding to the 2000 census. Exposure levels for each metal were calculated for each block group using the same methods as the tract level analysis. The resulting 50^th^ and 90^th^ percentiles of block group average levels were 1.24 and 2.75 ppb for arsenic, 0.50 and 1.90 ppb for cadmium, 41.85 and 163.00 ppb for manganese, and 3.32 and 7.55 ppb for lead. PRs and corresponding 95% CIs were calculated using log-linear regression as for the main analysis.

To further refine the exposure assessment, a secondary sensitivity analysis was conducted using a nested cohort of individuals residing outside of public water supply distribution areas. Using North Carolina public water supply area service data and the spatial overlay tool in ArcGIS, the nested analysis was limited to only those subjects with a geocoded maternal residence at delivery outside of a public water supply area. This selection restricted the analysis to individuals who are more likely to use private drinking water wells (n = 117,203 or 17% of the cohort included in the main analysis). PRs and corresponding 95% CIs were calculated identically to the main analysis using the tract and block group ecologic units.

## Results

### Study population characteristics

The final eligible study population for analysis included 20,151 case infants and 668,381 non-malformed control infants. Table 1 shows the distribution of demographic characteristics of the mothers and infants. Case and control subjects were similar with respect to age, race, sex, and maternal education status. There were a few notable differences by specific birth defect phenotype. Mothers of infants with anotia/microtia were more likely than controls to be Hispanic, and mothers of infants with gastroschisis were more likely to be young and have lower education compared to controls (data not shown). Prevalence of specific birth defects by maternal race/ethnicity has been reported previously by the North Carolina BDMP [34].

**Table 1:** **Characteristics of eligible case and control infants in the cohort**

Characteristic	Case infants (n = 20,151) Mean ± SD		Control infants (n = 668,381) Mean ± SD	
Maternal age at delivery	26.8 ± 6.2		26.9 ± 6.1	
*Missing*	*0*		*3*	
	n	(%)	n	(%)
Maternal race/ethnicity				
Non-Hispanic White	11,658	(57.9)	381,262	(57.0)
Non-Hispanic Black	4,751	(23.6)	152,370	(22.8)
Hispanic	2,976	(14.8)	106,003	(15.9)
Other^a^	745	(3.7)	28,009	(4.2)
*Missing*	*21*	*(<0.1)*	*737*	*(<0.1)*
Maternal education^b^				
< High school	5,035	(25.0)	151,732	(22.7)
High school	5,934	(29.4)	190,393	(28.5)
> High school	9,125	(45.3)	324,228	(48.5)
*Missing*	*57*	*(0.3)*	*2,028*	*(0.3)*
Infant sex				
Female	7,684	(38.1)	327,520	(49.0)
Male	12,463	(61.8)	340,858	(51.0)
*Missing*	*4*	*(0.2)*	*3*	*(<0.1)*

^a^Other race/ethnicity included individuals reporting Asian, and Pacific Islander/Native American race.

^b^Highest year of education completed.

### Metal levels elevated in some private wells

From the well water database records collected between 1998 and 2010, the analysis included between 11,000 and 47,000 wells with geocoded locations (See Methods; Additional file 1: Table S1). Notably, private well metal levels that exceeded the EPA regulatory guidelines for public drinking water were detected in 1,121 (2.4%) wells for arsenic, 16 (0.1%) wells for cadmium, 9,424 (20.5%) wells for manganese, and 1,434 (3.1%) wells for lead (Additional file 1: Table S1).

**Figure 1:**
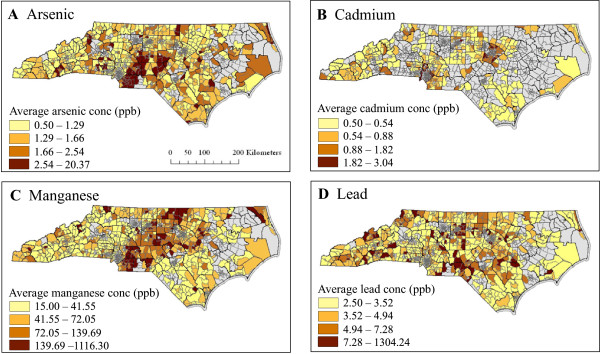
**Average levels of arsenic (A), cadmium (B), manganese (C), and lead (D) within census tracts across North Carolina.** The color gradient represents the calculated exposure percentile categories (≤50^th^; 50-75^th^; 75-90^th^; ≥90^th^ percentiles). The dark brown category represents exposed tracts and the light yellow category represents unexposed tracts that were used to assess the association with birth defects prevalence. Gray areas had fewer than 10 tested wells and were excluded from the analysis.

The geocoded levels of arsenic, cadmium, manganese, and lead in private wells were summarized to average census tract levels (Figure 1). The range of average tract levels was 0.5 to 20.4 ppb for arsenic, 0.5 to 3.0 ppb for cadmium, 15.0 to 1116.3 ppb for manganese, and 2.5 to 1304.2 ppb for lead. The average level of arsenic, manganese, or lead exceeded EPA drinking water guideline levels in at least one census tract (Additional file 1: Table S1). Notable trends of differential metal levels are evident across the state. Both arsenic and manganese levels were elevated around the central area of the state and these levels were correlated with each other (r = 0.21, p < 0.001 for tract-level correlation; Additional file 1: Table S2). Additionally, cadmium levels in wells were elevated in areas near cities (e.g. Raleigh, Charlotte), whereas lead levels appeared to be randomly spatially distributed, had a high coefficient of variation, and were not strongly correlated with other metals (Additional file 1: Table S2). The average levels of lead showed the greatest variation among tracts statewide compared to the variation of the average levels of manganese, arsenic or cadmium (Additional file 1: Table S2).

To examine the spatial occurrence of each birth defect category within census tracts, a dichotomous exposure status was assigned based on 50^th^ and 90^th^ percentiles of average concentration for each metal (see Methods). The patterns of these levels are represented in Figure 1. This dichotomous exposure assignment was developed to sharpen the exposure contrast by emphasizing the tails of the distribution of metal concentrations in our study area and was developed independently from EPA MCL guidelines for metals in public drinking water supplies. Table 2 reports the frequency of case and control infants among the exposed category for each metal. Overall, a similar proportion of case and control infants resided in exposed census tracts for each metal.

**Table 2:** **Frequency of exposed case and control infants where the exposed group is defined as living in a census tract with an average metal level greater than or equal to the 90** ^**th**^ **percentile** ^**a**^ **of average metal concentrations in tracts across the state**

	Arsenic n (%)^c^	Cadmium n (%)^c^	Manganese n (%)^c^	Lead n (%)^c^
1. Spina bifida (n = 218)	8 (3.7)	4 (1.8)	11 (5.0)	13 (6.0)
2. Anotia/microtia (n = 94)	3 (3.2)	3 (3.2)	6 (6.4)	5 (5.3)
3. Conotruncals (n = 435)	20 (4.6)	16 (3.7)	31 (7.1)	19 (4.4)
4. AVSD/ECD (n = 150)	8 (5.3)	5 (3.3)	10 (6.7)	5 (3.3)
5. HLHS (n = 142)	3 (2.1)	5 (3.5)	4 (2.8)	13 (9.2)
6. Cleft palate (n = 351)	17 (4.8)	15 (4.3)	18 (5.1)	20 (5.7)
7. Cleft lip ± CP (n = 516)	35 (6.8)	22 (4.3)	35 (6.8)	29 (5.6)
8. EA/TEF (n = 140)	9 (6.4)	6 (4.3)	7 (5.0)	8 (5.7)
9. Pyloric stenosis (n = 1,204)	73 (6.1)	20 (1.7)	63 (5.2)	59 (4.9)
10. Limb reduction (n = 255)	7 (2.7)	5 (2.0)	12 (4.7)	12 (4.7)
11. Gastroschisis (n = 215)	12 (5.6)	7 (3.3)	6 (2.8)	16 (7.4)
12. Hypospadias (n = 1,994)	92 (4.6)	94 (4.7)	107 (5.4)	122 (6.1)
Total cases (n = 5,534^b^)	283 (5.1)	196 (3.5)	303 (5.5)	310 (5.6)
Total controls (n = 668,381)	33,252 (5.0)	29,579 (4.4)	36,845 (5.5)	35,492 (5.3)

^a^The 90^th^ percentile of census tract average levels was 2.54 ppb for arsenic, 1.82 ppb for cadmium, 139.69 ppb for manganese, and 7.28 ppb for lead.

^b^Number of case infants with a diagnosis of one or more of the twelve prioritized structural outcomes of interest.

^c^Percent of each specific defect (Example: 8/218 = 3.7%).

*Abbreviations*: ‘Conotruncals’ consists of a group of heart defects including common truncus, Tetralogy of Fallot, and transposition of the great arteries; Atrioventricular septal/Endocardial cushion defects (AVSD/ECD); Hypoplastic left heart syndrome (HLHS); Esophageal atresia also called tracheo-esophageal fistula (EA/TEF); ‘Limb reduction’ includes reduction defects of the upper and lower limbs.

Crude and adjusted PRs and 95% CIs were calculated to estimate the association between average concentration of each metal and the prevalence of birth defects within census tracts (Additional file 1: Table S3; Table 3). Table 3 reports the adjusted PR estimates comparing the prevalence of birth defects between the metal-exposed versus unexposed tracts. To facilitate the interpretation of results, this study considered positive effect measures >1.3 or for which the 95% CI exclude the null (1.0) to be noteworthy potential metal-defect associations. The arsenic-exposed category was associated with a statistically significantly decreased prevalence of HLHS (PR: 0.3 95% CI: 0.1-1.0) compared to the unexposed category. Two additional notable elevated PRs were observed for arsenic-exposed versus unexposed and the prevalence of cleft lip with or without cleft palate (PR: 1.3 95% CI: 0.9-1.9) and EA/TEF (PR: 1.3 95% CI: 0.4-4.3), but the observed 95% CIs were consistent with a null result.

**Table 3:** **Association** ^**a**^ **[PR (95% CI)] between selected metals and birth defects**

Defect	Arsenic	Cadmium	Manganese	Lead
1. Spina bifida	0.7 (0.3, 1.4)	0.5 (0.2, 1.4)	0.7 (0.4, 1.4)	1.0 (0.5, 1.7)
2. Anotia/microtia	0.6 (0.2, 2.0)	1.0 (0.3, 4.2)	1.3 (0.5, 3.4)	1.0 (0.4, 2.6)
3. Conotruncals	1.0 (0.6, 1.6)	1.1 (0.6, 2.0)	1.6 (1.1, 2.5)*	0.9 (0.6, 1.5)
4. AVSD/ECD	1.1 (0.5, 2.3)	0.7 (0.2, 1.9)	1.3 (0.6, 2.5)	0.7 (0.3, 1.8)
5. HLHS	0.3 (0.1, 1.0)*	0.8 (0.3, 2.4)	0.5 (0.2, 1.3)	1.7 (0.9, 3.3)
6. Cleft palate (CP)	0.8 (0.5, 1.3)	1.1 (0.6, 2.1)	0.7 (0.5, 1.2)	0.9 (0.6, 1.5)
7. Cleft lip ± CP	1.3 (0.9, 1.9)	1.0 (0.6, 1.7)	1.2 (0.9, 1.8)	1.0 (0.7, 1.5)
8. EA/TEF	1.3 (0.6, 2.7)	0.9 (0.3, 2.4)	0.9 (0.4, 2.0)	1.1 (0.5, 2.3)
9. Pyloric stenosis	1.1 (0.9, 1.5)	0.4 (0.3, 0.7)*	0.8 (0.6, 1.1)	0.8 (0.6, 1.1)
10. Limb reduction	0.5 (0.2, 1.0)	0.6 (0.2, 1.6)	0.7 (0.4, 1.2)	0.7 (0.4, 1.4)
11. Gastroschisis	1.0 (0.5, 1.8)	1.2 (0.5, 2.8)	0.5 (0.2, 1.1)	1.4 (0.8, 2.4)
12. Hypospadias	0.9 (0.7, 1.1)	0.9 (0.7, 1.2)	0.9 (0.7, 1.1)	1.1 (0.9, 1.4)

^a^Prevalence ratios were adjusted for maternal age, race, and education status. Metal exposure status was dichotomized as ≥90^th^ percentile of average census tract metal levels or ≤50^th^ percentile. Where the 50^th^ and 90^th^ percentile of census tract average levels were as follows: 1.29 and 2.54 ppb for arsenic, 0.54 and 1.82 ppb for cadmium, 41.55 and 139.69 ppb for manganese, and 3.52 and 7.28 ppb for lead.

*p < 0.05.

*Abbreviations*: ‘Conotruncals’ consists of a group of heart defects including common truncus, Tetralogy of Fallot, and transposition of the great arteries; Atrioventricular septal/Endocardial cushion defects (AVSD/ECD); Hypoplastic left heart syndrome (HLHS); Esophageal atresia also called tracheo-esophageal fistula (EA/TEF); ‘Limb reduction’ includes reduction defects of the upper and lower limbs.

The manganese-exposed category was statistically significantly associated with a higher prevalence of conotruncal heart defects (PR: 1.6 95% CI: 1.1-2.5) compared to the unexposed category. An elevated PR was also observed for manganese-exposed versus unexposed categories and the prevalence of anotia/microtia (PR: 1.3 95% CI: 0.5-3.4) and AVSD/ECD (PR: 1.3 95% CI: 0.6-2.5), however the CIs were consistent with unity.

The lead-exposed category was associated with an increased prevalence of HLHS (PR: 1.7 95% CI: 0.9-3.3), but the finding was not statistically significant. The cadmium-exposed group was associated with a significantly decreased prevalence of pyloric stenosis (PR: 0.4 95% CI: 0.3-0.7) compared to the unexposed group. The findings did not suggest patterns of association with cadmium-exposure and an increased prevalence of any birth defect analyzed in this study.

To account for multiple comparisons, Bonferroni correction was applied and 99.9% CIs are reported (Additional file 1: Table S4). The inverse association between cadmium exposure and pyloric stenosis was the only observed association that remained statistically significant after Bonferroni correction (PR: 0.4 99.9% CI: 0.2-1.0).

The potential interaction between joint exposure to the exposed categories of arsenic and manganese and the prevalence of each birth defect was examined. Adjusted PR estimates and 95% CIs showed elevated (though not statistically significant) point estimates for the interaction effect of arsenic and manganese exposure with defect outcomes including anotia/microtia (PR: 2.7 95% CI: 0.7-10.2), conotruncal defects (PR: 1.6 95% CI: 0.8-3.2), AVSD/ECD (PR: 1.5 95% CI: 0.5-4.3) and cleft lip with or without cleft palate (PR: 1.4 95% CI: 0.8-2.6) (Additional file 1: Table S5).

### Sensitivity analyses results

**Figure 2:**
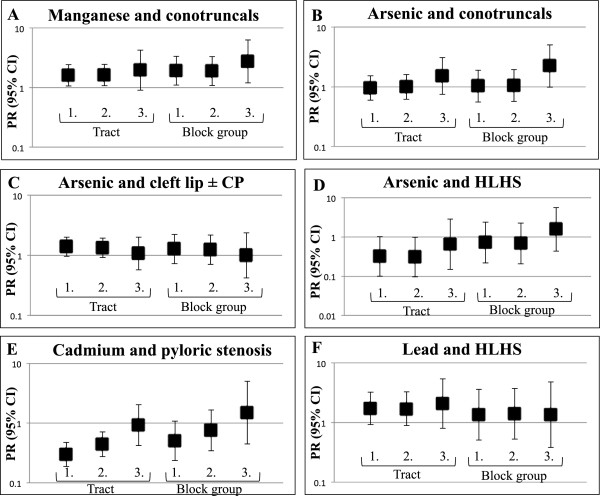
**Sensitivity analysis plots for selected associations between residence in ecologic units of highest compared to lowest metal level categories and prevalence of birth defects including the following metal-defect pairs: A) manganese and conotruncal heart defects, B) arsenic and conotruncal heart defects, and C) arsenic and cleft lip with or without cleft palate (CP), D) arsenic and hypoplastic left heart syndrome (HLHS), E) cadmium and pyloric stenosis, and F) lead and hypoplastic left heart syndrome (HLHS).** Prevalence ratios (PRs) and 95% confidence intervals (CIs) correspond as follows: 1. Crude estimate, 2. Adjusted estimate for maternal age, race and education status, or 3. Estimate restricted to individuals outside public service areas and adjusted for maternal age, race and education status.

To assess the impact of potential exposure misclassification in the ecologic analysis, two sensitivity analyses were conducted (Additional file 1: Tables S6-S8). For both types of sensitivity analyses, the PR estimates had wider CIs due to the reduction in sample size. The PRs and 95% CIs for selected relationships assessed in the sensitivity analyses are shown in Figure 2. The findings suggest patterns of association consistent with the highest category of manganese exposure and conotruncal heart defects (Figure 2A). For example, the association between manganese exposure and conotruncal heart defects was estimated under six conditions including the tract ecologic unit crude PR (95% CIs) of 1.6 (1.1-2.4) (Additional file 1: Table S3) and adjusted PR (95% CI) of 1.6 (1.1-2.5) (Table 1), which increased to 2.0 (0.9-4.3) (Additional file 1: Table S6) when restricted to individuals outside of public supply areas. The same estimates using the block group ecologic unit estimated a crude PR (95% CI) of 1.9 (1.1-3.4), an adjusted PR (95% CI) of 1.9 (1.1-3.4) (Additional file 1: Table S7), which increased to 2.8 (1.2-6.4) (Additional file 1: Table S8) when restricted to individuals outside of public supply areas. Likewise, the association between arsenic exposure and conotruncal heart defects showed a suggestive increasing trend in estimates (Figure 2B; Additional file 1: Tables S6-S8). Other metal-defect associations, such as the relationships between arsenic and cleft lip, arsenic and HLHS, or cadmium and pyloric stenosis, or lead and HLHS, however, show trends suggestive of a null relationship (Figure 2C-F).

## Discussion

In the present study a statewide semi-ecologic analysis was conducted to investigate the potential association between metal concentrations in private well water and birth defects prevalence in North Carolina. Specifically, we examined the association between the prevalence of twelve birth defect groups and levels of arsenic, cadmium, manganese, and lead summarized at the census tract ecologic unit. Taken together, our analyses identified three notable findings: (i) metal levels are elevated in private wells across the state and some metals such as arsenic and manganese co-occur, (ii) residence in manganese-exposed census tracts was statistically significantly associated with increased prevalence of conotruncal heart defects, and (iii) there is suggestive evidence of an interactive effect between residence in arsenic- and manganese-exposed census tracts and the prevalence of conotruncal heart defects.

### Elevated environmental metals are spatially correlated in private wells

North Carolina is the fourth largest state population with approximately 2.3 million people relying on private wells for drinking water; the total number of individuals served is larger only in Pennsylvania, California, and Michigan as estimated by the USGS [27]. Arsenic has been previously recognized as a contaminant of concern in certain regions of North Carolina [30–32]. The present study extends our previous findings reported in [30] to identify census tract and block group levels of average arsenic, manganese, and lead that exceeded respective EPA standards for public distribution systems, and to map spatial trends of these metals in wells across the state.

The levels of metals in private wells is not currently regulated in North Carolina, and routine testing of private well water quality for contaminants is not required by state or federal regulations (state regulation requires a single test only when new wells are constructed). The MCL for arsenic levels in public water supplies is 10 μg/L, and 5 μg/L for cadmium [35]. Lead is regulated according to a Treatment Technique (TT) designed to control corrosiveness that requires that no more than 10% of a system’s water samples measured at consumer taps can exceed 15 μg/L [35]. Manganese has a recommended Secondary Maximum Contaminant Level (SMCL) of 50 μg/L in public water supplies that is based on taste and odor and is not federally enforceable [35]. Based on these standards, 1,121 (2.4%) of arsenic, 16 (0.1%) of cadmium, 1,434 (3.1%) of lead, and 9,424 (20.5%) of manganese samples from individual wells tested between 1998 and 2010 and included in our study exceeded guideline levels. The extent of samples exceeding the SMCL for manganese is substantial, and comparable to a similar proportion of wells (~21%) that exceeded 50 μg/L in a nationwide study [36]. The arsenic data are supported by our previous findings that demonstrated arsenic contamination in areas along a geographic area known as the Carolina terrane [30]. Levels of cadmium or lead in private wells in North Carolina have not been previously reported.

Spatial trends of private well metal contamination were identified across North Carolina. Here we find that manganese levels correlate spatially with arsenic levels in private wells. Levels of arsenic and manganese have been described in North Carolina groundwater [29–32]; however, a spatial correlation has not been previously reported with respect to private well levels. Elevated cadmium levels were evident near major cities including Raleigh and Charlotte. Urbanization and anthropogenic activities are contributing factors to groundwater contamination from metals including cadmium and arsenic [37]. Lead can occur at elevated levels in drinking water by leaching from certain faucets, fittings, or water systems [38], which may explain the random spatial appearance of levels across the state. Despite the lack of state and federal regulation, it is recommended that individuals relying on private wells for drinking water have their water quality tested annually.

### Manganese levels associated with heart defects prevalence

Our findings support a potential association between residence in manganese-exposed census tracts and the prevalence of conotruncal heart defects. The results of the sensitivity analyses showed similar positive trends and statistically significant associations that are suggestive of a potential association between manganese levels and conotruncal defects. To our knowledge, to date no studies have examined the relationship between drinking water manganese and birth defects in humans. A previous study reported that increased placental manganese levels were associated a higher risk of NTDs showed a statistically significant association with spina bifida [39]. In animals, both increased and decreased levels of manganese have been associated with teratogenicity (reviewed in [40]). Furthermore, manganese exposure has been associated with several adverse newborn outcomes including infant mortality [29, 41], intrauterine growth restriction [42], and lower birth weight [11, 43]. As manganese can cross the placental barrier and bioaccumulate in fetal and neonatal tissue [5, 7, 44, 45], the potential association between manganese and birth defects is biologically plausible. Manganese exposure is associated with adverse cardiovascular and central nervous system effects in adult populations [3, 46]; however, its role in abnormal prenatal cardiac development requires further study. Dietary, occupational, and industrial exposures, as well as inhalation of manganese aerosols during showering remain important considerations that contribute to total manganese intake in addition to drinking water [3, 47, 48]. These data suggest that additional studies examining the health effects of manganese as a developmental toxicant with potential exposure from drinking water are warranted.

### Arsenic levels were modestly associated with birth defects prevalence

The results suggest that residence within arsenic-exposed census tracts was modestly associated with a higher prevalence of cleft lip with or without cleft palate and EA/TEF defects at the census tract ecologic unit. Residence in arsenic-exposed tracts was also significantly associated with decreased prevalence of HLHS at the census tract ecologic unit, although the sensitivity analyses were consistent with a trend towards a null association. Previous population-based studies have shown associations between drinking water arsenic levels and CHDs [19–21], NTDs [22], and increased prevalence of all birth defects [49]. It should be noted that for two of these studies [21, 22], although the point estimates were elevated in relation to higher concentrations of arsenic in drinking water, the 95% CIs were consistent with the null. While there is strong evidence of the teratogenic effects of arsenic compounds in animal models, currently inorganic arsenic teratogenicity in humans remains debated [24, 26].

### Arsenic-by-manganese interaction

Our results show a potential interactive effect between arsenic and manganese on the association with spina bifida, conotruncal heart defects, AVSD heart defects, and cleft lip with or without cleft palate. Specifically, the estimates reveal that the prevalence of these defects among infants residing in census tracts that were considered both arsenic- and manganese-exposed compared to areas that were arsenic- and manganese-unexposed were increased compared to the PR estimates observed for either metal alone. The findings suggest a potential, but not statistically significant, joint effect in areas of arsenic and manganese co-occurrence. These analyses had limited power as reflected in the width of the reported CIs because the total sample size was considerably reduced. This association has not been previously examined in studies of environmental metal exposures and birth defects. There is previous evidence for potential drinking water co-exposure to both manganese and arsenic. In a study of US public wells, the combination of arsenic and manganese detected in the same water source was one of the most commonly occurring pairs of unique mixtures out of over 90 organic and inorganic contaminants that were tested [50].

### Cadmium and lead levels revealed reduced or null associations

The results suggest some associations between residence in areas of the highest cadmium levels and reduced birth defects prevalence. A decreased prevalence in pyloric stenosis was associated with residence in areas of the highest cadmium exposure, a relationship that remained statistically significant after Bonferroni correction. There were no statistically significant positive relationships between birth defect prevalence and residence in areas of the highest cadmium or lead levels. At the time of this study, we are aware of only three studies that examined the relationship between drinking water levels of cadmium and/or lead and birth defects in humans [21, 22, 51]. Two studies reported null associations between drinking water levels and NTDs [22, 51], and the other reported an increased odds of heart defects associated with detectable lead levels in drinking water [21]. Blood lead levels or occupational lead exposure have been associated with CHDs [52, 53], NTDs [54, 55], and cleft lip [55, 56]. Further, animal studies confirm cadmium- and lead-induced malformations in animal models [57–60]. While counterintuitive, there is evidence for protective effects of maternal cigarette smoking, a major source of cadmium, and orofacial clefts, NTDs and conotruncal heart defects [61], but an inverse relationship with pyloric stenosis prevalence has not been previously reported. The present study could not account for maternal smoking status due to limited sample size, and this may represent a source of unmeasured confounding for some birth defect phenotypes.

### Study limitations and strengths

This study had several limitations that are important when interpreting the results. As individual-level environmental data were not available within the birth defects database, it was not possible to evaluate the relationship between personal water consumption patterns and the risk of defects. Data on individual use of well water were unavailable; therefore, any observed associations are limited to the ecologic unit of analysis and the findings are subject to the ecologic fallacy. However, in the absence of individual-level exposure data, ecologic approaches have previously been applied to examine associations between metals in private wells and other health outcomes [62, 63].

Exposure assessment remains a challenge among epidemiological studies of birth defects [64, 65]. For example, there are no existing biomonitoring programs in North Carolina to actively assess environmental exposures during the prenatal period. As a result, research paradigms usually require retrospective exposure assessment that is clearly limited by issues of temporality. Our recent study suggests that prenatal biomonitoring may be warranted in North Carolina [66], especially among at-risk populations. Such a biomonitoring program would enable a direct prospective assessment of the relationship between environmental exposures and children born with birth defects.

In this study the assigned exposures represent a census tract average over a period of 12 years and BDMP data represent the location of cases at delivery. It is possible that non-differential exposure misclassification was introduced by the databases including a potentially biased sample of tested wells, a large proportion of tracts with limited sampling, and the likelihood of maternal mobility during pregnancy [67]. It is possible that bias may have been introduced by the private well database since it represents a non-random sample of wells for which the owners requested metal analyses. Similarly, the state testing system does not assign unique well IDs, and some wells likely were tested more than once over the 11-year period. There may also be effects of residual confounding related to geographical location such as socioeconomic status and/or residence in rural versus urban areas that were not accounted for in the analysis. We attempt to address some of these limitations by refining exposure assessment, at the expense of statistical precision, by applying two sensitivity analyses. A sensitivity analysis removed individuals residing in areas served by public water supplies. This resulted in approximately 17% of the initial cohort size. In preliminary data from the National Birth Defects Prevention Study (NBDPS), roughly 22% of mothers residing a subset of 19 North Carolina counties over a similar time period reported having a private well tap water source (data not shown). We interpret the NBDPS data with caution, as the subset of 19 counties may not reflect the appropriate proportion of private well users statewide. Given the lack of individual-level data, this ecologic method served as an imperfect proxy for type of water supply as it potentially excludes a fraction of individuals who use private wells but may be within the public distribution service area. Overall, the results of the sensitivity analyses suggest some metal-defect trends. PR estimates using the block group unit and when restricted to individuals outside of public water utility areas were elevated suggesting that exposure misclassification at the tract level may have biased associations towards the null. We note that in both sensitivity analyses, the wide CIs reflect a loss of precision due to decreased sample sizes.

This study had several strengths including an active statewide case ascertainment program, which provided a large sample size of liveborn cases and controls representing six years of active birth defects surveillance in North Carolina. The semi-ecologic design enabled individual-level assessment of newborns with specific defects and covariate adjustment using data from birth certificates. The outcomes we investigated included specific phenotypes or groups of phenotypes based on similar etiologic mechanisms, which is advantageous over previous studies that have grouped multiple defect outcomes. The statewide surveillance program allowed exclusion of case infants born with chromosomal abnormalities, as this is a known risk factor for some structural defects [68]. This study is the first to assess the association between toxic metal levels in drinking water and birth defects in North Carolina, and builds on a limited body of existing literature on the potential relation between toxic metals and risk of birth defects.

## Conclusions

Overall, our findings suggest evidence at the ecologic level of possible relationships between elevated concentrations of arsenic, cadmium, manganese, and lead levels in drinking water and specific birth defects. Given that our results support previous evidence of an association between metal exposure and specific heart defects, these relationships should be prioritized for future studies. This is directly in line with ongoing research directed at understanding the biological mechanisms that underlie metal-birth defect associations using animal and cell culture models [69]. Further studies are necessary to validate the potential associations observed in this study and to better understand the complex environmental, genomic, and epigenetic mechanisms involved in potentially preventable environmentally-mediated birth defects.

## Electronic supplementary material

Additional file 1: Table S1: Number of geocoded wells for each metal as well as number of census tract and block groups with average metal levels above EPA drinking water standards for public distribution systems. Averages were calculated where more than ten wells were measured in the respected block group or tract. **Table S2.** Spearman’s rank correlations and coefficient of variation for average (ppb) census tract metal levels and census block group metal levels (noted in parentheses). **Table S3.** Crude PRs and 95% CIs using the census tract ecologic unit and comparing individuals in the exposed (≥90^th^ percentile) to unexposed (≤50^th^ percentile) groups for average census tract metal levels. **Table S4.** Bonferroni corrected adjusted PRs and 99.9% CIs using the census tract ecologic unit and comparing individuals in the exposed (≥90^th^ percentile) to unexposed (≤50^th^ percentile) groups for average census tract metal levels. PRs were adjusted for maternal age, race, and education status. **Table S5.** Crude and adjusted PRs and 95% CIs estimate arsenic-by-manganese interaction using the tract level ecologic unit. PRs were adjusted for maternal age, race, and education status. **Table S6.** Adjusted PRs and 95% CIs using the census tract ecologic unit and excluding individuals living in public distribution areas. PRs were adjusted for maternal age, race, and education status. **Table S7.** Crude and adjusted PRs and 95% CIs using the block group ecologic unit and comparing individuals in the exposed (≥90^th^ percentile) to unexposed (≤50^th^ percentile) groups for average census block group metal levels. PRs were adjusted for maternal age, race, and education status. **Table S8.** Adjusted PRs and CIs at the block group level excluding individuals living in public distribution areas. PRs were adjusted for maternal age, race, and education status. (DOCX 35 KB)

## Abbreviations

AVSD: Atrioventricular septal defect
CHD: Congenital heart defect
CI: Confidence interval
CP: Cleft palate
DHHS: Department of Health and Human Services
EA: Esophageal atresia
ECD: Endocardial cushion defect
HLHS: Hypoplastic left heart syndrome
MCL: Maximum Contaminant Level
NTD: Neural tube defect
PR: Prevalence ratio
SMCL: Secondary maximum contaminant level
TEF: Tracheo-esophageal fistula
TOF: Tetralogy of fallot
TGA: Transposition of the great arteries
TT: Treatment technique.
