# Supplementary material for: Association between arsenic, cadmium, manganese, and lead levels in private wells and birth defects prevalence in North Carolina: a semi-ecologic study
Source: BMC Public Health. 2014 Sep 15;14:955. doi: 10.1186/1471-2458-14-955 (PMC4190372; doi:10.1186/1471-2458-14-955)
Supplement: Supplementary file 1 — Additional file 1: Table S1: Number of geocoded wells for each metal as well as number of census tract and block groups with average metal levels above EPA drinking water standards for public distribution systems. Averages were calculated where more than ten wells were measured in the respected block group or tract. Table S2. Spearman’s rank correlations and coefficient of variation for average (ppb) census tract metal levels and census block group metal levels (noted in parentheses). Table S3. Crude PRs and 95% CIs using the census tract ecologic unit and comparing individuals in the exposed (≥90th percentile) to unexposed (≤50th percentile) groups for average census tract metal levels. Table S4. Bonferroni corrected adjusted PRs and 99.9% CIs using the census tract ecologic unit and comparing individuals in the exposed (≥90th percentile) to unexposed (≤50th percentile) groups for average census tract metal levels. PRs were adjusted for maternal age, race, and education status. Table S5. Crude and adjusted PRs and 95% CIs estimate arsenic-by-manganese interaction using the tract level ecologic unit. PRs were adjusted for maternal age, race, and education status. Table S6. Adjusted PRs and 95% CIs using the census tract ecologic unit and excluding individuals living in public distribution areas. PRs were adjusted for maternal age, race, and education status. Table S7. Crude and adjusted PRs and 95% CIs using the block group ecologic unit and comparing individuals in the exposed (≥90th percentile) to unexposed (≤50th percentile) groups for average census block group metal levels. PRs were adjusted for maternal age, race, and education status. Table S8. Adjusted PRs and CIs at the block group level excluding individuals living in public distribution areas. PRs were adjusted for maternal age, race, and education status. (DOCX 35 KB) [file 12889_2014_7097_MOESM1_ESM.docx]

**Additional file 1**

**Supplemental Tables.**

Table S1. Number of geocoded wells for each metal as well as number of census tract and block groups with average metal levels above EPA drinking water standards for public distribution systems. Averages were calculated where more than ten wells were measured in the respected block group or tract.

|  | Total number of geocoded wells | Individual wells | Tracts exceeding EPA drinking water standard (n_Total_=1,563) | Block Groups exceeding  EPA drinking water standard (n_Total_=5,271) | EPA reference level |
| --- | --- | --- | --- | --- | --- |
| As | 46,286 | 1,121 (2.4%) | 13 (0.8%) | 42 (0.8%) | 10 ppb |
| Cd | 11,381 | 16 (0.1%) | 0 (0.0%) | 0 (0.0%) | 5 ppb |
| Mn | 46,022 | 9,424 (20.5%) | 308 (19.7%) | 538 (10.2%) | 50 ppb |
| Pb | 46,059 | 1,434 (3.1%) | 27 (1.7%) | 46 (0.9%) | 15 ppb |

Table S2. Spearman’s rank correlations and coefficient of variation for average (ppb) census tract metal levels and census block group metal levels (noted in parentheses).

|  | Spearman’s Rank Correlation | | | Coefficient of Variation |
| --- | --- | --- | --- | --- |
|  | Cd | Mn | Pb |  |
| As | -0.17* (-0.19**) | 0.21** (0.24**) | -0.03 (-0.01) | 119.95 (165.81) |
| Cd |  | -0.04 (0.05) | 0.10 (0.07) | 63.26 (65.81) |
| Mn |  |  | 0.10* (0.05) | 128.14 (192.55) |
| Pb |  |  |  | 665.13 (878.59) |

*p<0.05; **p<0.001

Table S3. Crude PRs and 95% CIs using the census tract ecologic unit and comparing individuals in the exposed (≥90^th^ percentile) to unexposed (≤50^th^ percentile) groups for average census tract metal levels.

| Defect | As | Cd | Mn | Pb |
| --- | --- | --- | --- | --- |
| 1. Spina bifida | 0.7 (0.3, 1.4) | 0.4 (0.1, 1.2) | 0.7 (0.4, 1.4) | 1.0 (0.5, 1.8) |
| 2. Anotia/ microtia | 0.6 (0.2, 2.1) | 1.1 (0.3, 4.2) | 1.4 (0.6, 3.4) | 1.0 (0.4, 2.7) |
| 3. Conotruncals | 1.0 (0.6, 1.6) | 1.0 (0.6, 1.9) | 1.6 (1.1, 2.4)* | 0.9 (0.6, 1.5) |
| 4. AVSD/ ECD | 1.1 (0.5, 2.3) | 0.6 (0.2, 1.6) | 1.2 (0.6, 2.5) | 0.7 (0.3, 1.8) |
| 5. HLHS | 0.3 (0.1, 1.0)* | 0.7 (0.2, 1.8) | 0.4 (0.2, 1.3) | 1.7 (0.9, 3.3) |
| 6. Cleft palate | 0.8 (0.5, 1.4) | 1.0 (0.5, 1.8) | 0.7 (0.5, 1.2) | 0.9 (0.6, 1.5) |
| 7. Cleft lip ± CP | 1.4 (1.0, 2.0) | 0.9 (0.5, 1.4) | 1.2 (0.8, 1.8) | 1.0 (0.7, 1.5) |
| 8. EA/ TEF | 1.3 (0.6, 2.7) | 1.0 (0.4, 2.6) | 0.9 (0.4, 2.0) | 1.1 (0.5, 2.3) |
| 9. Pyloric stenosis | 1.3 (1.0, 1.6) | 0.3 (0.2, 0.5)* | 0.8 (0.6, 1.1) | 0.8 (0.6, 1.1) |
| 10. Limb reduction | 0.5 (0.2, 1.1) | 0.5 (0.2, 1.3) | 0.7 (0.4, 1.2) | 0.8 (0.5, 1.5) |
| 11. Gastroschisis | 1.2 (0.6, 2.2) | 0.6 (0.3, 1.4) | 0.4 (0.2, 1.0) | 1.4 (0.8, 2.5) |
| 12. Hypospadias | 0.8 (0.7, 1.1) | 0.9 (0.7, 1.2) | 0.9 (0.7, 1.1) | 1.1 (0.9, 1.3) |

*p<0.05

Table S4. Bonferroni corrected adjusted PRs and 99.9% CIs using the census tract ecologic unit and comparing individuals in the exposed (≥90^th^ percentile) to unexposed (≤50^th^ percentile) groups for average census tract metal levels. PRs were adjusted for maternal age, race, and education status.

| Defect | As | Cd | Mn | Pb |
| --- | --- | --- | --- | --- |
| 1. Spina bifida | 0.7 (0.2, 2.3) | 0.5 (0.1, 3.0) | 0.7 (0.3, 2.1) | 1.0 (0.4, 2.6) |
| 2. Anotia/ microtia | 0.6 (0.1, 4.5) | 1.0 (0.1, 10.9) | 1.3 (0.3, 6.2) | 1.0 (0.2, 4.9) |
| 3. Conotruncals | 1.0 (0.5, 2.2) | 1.1 (0.4, 3.0) | 1.6 (0.8, 3.3) | 0.9 (0.4, 2.1) |
| 4. AVSD/ ECD | 1.1 (0.3, 3.8) | 0.7 (0.1, 3.8) | 1.3 (0.4, 4.1) | 0.7 (0.1, 3.3) |
| 5. HLHS | 0.3 (0.0, 2.2) | 0.8 (0.1, 4.8) | 0.5 (0.1, 2.6) | 1.7 (0.6, 5.0) |
| 6. Cleft palate | 0.8 (0.3, 1.9) | 1.1 (0.4, 3.2) | 0.7 (0.3, 1.7) | 0.9 (0.4, 2.1) |
| 7. Cleft lip ± CP | 1.3 (0.7, 2.5) | 1.0 (0.4, 2.3) | 1.2 (0.7, 2.3) | 1.0 (0.5, 1.9) |
| 8. EA/ TEF | 1.3 (0.4, 4.3) | 0.9 (0.2, 4.8) | 0.9 (0.2, 3.4) | 1.1 (0.3, 3.9) |
| 9. Pyloric stenosis | 1.1 (0.7, 1.8) | 0.4 (0.2, 1.0)* | 0.8 (0.5, 1.3) | 0.8 (0.5, 1.3) |
| 10. Limb reduction | 0.5 (0.1, 1.8) | 0.6 (0.1, 3.1) | 0.7 (0.2, 1.9) | 0.7 (0.3, 2.2) |
| 11. Gastroschisis | 1.0 (0.3, 2.8) | 1.2 (0.3, 5.1) | 0.5 (0.1, 1.9) | 1.4 (0.5, 3.5) |
| 12. Hypospadias | 0.9 (0.6, 1.2) | 0.9 (0.6, 1.4) | 0.9 (0.6, 1.3) | 1.1 (0.8, 1.5) |

*p<0.05

Table S5. Crude and adjusted PRs and 95% CIs estimate arsenic-by-manganese interaction using the tract level ecologic unit. PRs were adjusted for maternal age, race, and education status.

| Defect | As*Mn Crude | As*Mn Adjusted |
| --- | --- | --- |
| 1. Spina bifida | 0.6 (0.2, 1.9) | 0.6 (0.2, 1.8) |
| 2. Anotia/ microtia | 2.9 (0.8, 10.6) | 2.7 (0.7, 10.2) |
| 3. Conotruncals | 1.6 (0.8, 3.2) | 1.6 (0.8, 3.2) |
| 4. AVSD/ ECD | 1.6 (0.6, 4.8) | 1.5 (0.5, 4.3) |
| 5. HLHS | 0.4 (0.1, 2.8) | 0.4 (0.0, 2.7) |
| 6. Cleft palate | 0.7 (0.3, 1.7) | 0.7 (0.3, 1.7) |
| 7. Cleft lip ± CP | 1.5 (0.9, 2.8) | 1.4 (0.8, 2.6) |
| 8. EA/ TEF | 1.2 (0.4, 3.9) | 1.2 (0.3, 3.9) |
| 9. Pyloric stenosis | 1.3 (0.9, 1.9) | 1.2 (0.8, 1.8) |
| 10. Limb reduction | 0.2 (0.0, 1.3) | 0.2 (0.0, 1.2) |
| 11. Gastroschisis | 0.5 (0.1, 2.1) | 0.5 (0.1, 1.9) |
| 12. Hypospadias | 0.7 (0.5, 1.1) | 0.7 (0.5, 1.1) |

Table S6. Adjusted PRs and 95% CIs using the census tract ecologic unit and excluding individuals living in public distribution areas. PRs were adjusted for maternal age, race, and education status.

| Defect | As | Cd | Mn | Pb |
| --- | --- | --- | --- | --- |
| 1. Spina bifida | 0.5 (0.1, 2.1) | NE | 0.7 (0.2, 2.2) | 1.3 (0.6, 3.1) |
| 2. Anotia/ microtia | NE | NE | NE | 1.8 (0.5, 6.8) |
| 3. Conotruncals | 1.5 (0.8, 3.1) | NE | 2.0 (0.9, 4.3) | 1.4 (0.7, 3.2) |
| 4. AVSD/ ECD | 1.1 (0.3, 3.9) | 0.8 (0.1, 6.1) | NE | 0.9 (0.2, 4.0) |
| 5. HLHS | 0.7 (0.2, 2.9) | 1.3 (0.2, 10.8) | 0.7 (0.2, 3.3) | 2.1 (0.8, 5.5) |
| 6. Cleft palate | 0.8 (0.4, 1.8) | 0.5 (0.1, 3.9) | 0.3 (0.1, 1.1) | 0.8 (0.3, 2.0) |
| 7. Cleft lip ± CP | 1.1 (0.6, 2.0) | 1.3 (0.4, 3.8) | 0.5 (0.2, 1.5) | 0.9 (0.4, 2.0) |
| 8. EA/ TEF | 1.2 (0.4, 3.4) | NE | 1.3 (0.4, 4.8) | 1.2 (0.3, 4.3) |
| 9. Pyloric stenosis | 1.1 (0.7, 1.7) | 0.9 (0.4, 2.0) | 1.0 (0.6, 1.6) | 1.2 (0.7, 1.8) |
| 10. Limb reduction | 0.3 (0.1, 1.5) | NE | 0.6 (0.2, 1.9) | 0.5 (0.1, 2.0) |
| 11. Gastroschisis | 1.3 (0.6, 3.0) | 0.8 (0.1, 6.1) | 0.9 (0.4, 2.4) | 1.3 (0.5, 2.9) |
| 12. Hypospadias | 0.8 (0.5, 1.2) | 1.4 (0.9, 2.2) | 0.8 (0.6, 1.2) | 1.1 (0.8, 1.6) |

NE -Not estimated.

Table S7. Crude and adjusted PRs and 95% CIs using the block group ecologic unit and comparing individuals in the exposed (≥90^th^ percentile) to unexposed (≤50^th^ percentile) groups for average census block group metal levels. PRs were adjusted for maternal age, race, and education status.

| Defect | As | Cd | Mn | Pb |
| --- | --- | --- | --- | --- |
|  | Crude/  Adjusted PRs | | | |
| 1. Spina bifida | 0.7 (0.3, 1.9)/  0.7 (0.3, 1.8) | 0.5 (0.1, 4.4)/ 0.6 (0.1, 5.8) | 1.7 (0.8, 3.7)/ 1.7 (0.8, 3.5) | 1.7 (0.8, 3.6)/ 1.6 (0.8, 3.5) |
| 2. Anotia/ microtia | 1.0 (0.3, 3.5)/ 0.9 (0.3, 3.2) | 1.3 (0.1, 14.0)/ NE | 0.7 (0.2, 3.2)/ 0.7 (0.2, 3.1) | NE/ NE |
| 3. Conotruncals | 1.0 (0.6, 1.9)/ 1.1 (0.6, 2.0) | 1.4 (0.5, 3.4)/ 1.5 (0.5, 3.9) | 1.9 (1.1, 3.4)*/ 1.9 (1.1, 3.4)* | 1.2 (0.6, 2.3)/ 1.2 (0.6, 2.3) |
| 4. AVSD/ ECD | 1.8 (0.7, 5.0)/ 1.7 (0.6, 4.5) | NE / NE | 1.8 (0.7, 5.1)/ 1.9 (0.7, 5.4) | 1.3 (0.4, 4.4)/ 1.4 (0.4, 4.8) |
| 5. HLHS | 0.7 (0.2, 2.4)/ 0.7 (0.2, 2.3) | NE / NE | 0.5 (0.1, 2.1)/ 0.5 (0.1, 2.1) | 1.4 (0.5, 3.6)/ 1.4 (0.5, 3.8) |
| 6. Cleft palate | 0.8 (0.4, 1.7)/ 0.8 (0.4, 1.7) | 0.5 (0.1, 2.3)/ 0.6 (0.1, 2.7) | 0.6 (0.3, 1.4)/ 0.6 (0.3, 1.4) | 1.0 (0.5, 1.8)/ 1.0 (0.5, 1.9) |
| 7. Cleft lip ± CP | 1.3 (0.7, 2.2)/ 1.2 (0.7, 2.2) | 0.8 (0.3, 2.1)/ 1.0 (0.4, 2.6) | 1.0 (0.5, 1.9)/ 1.0 (0.5, 1.9) | 1.2 (0.7, 2.2)/ 1.2 (0.7, 2.2) |
| 8. EA/ TEF | 1.2 (0.5, 3.2)/ 1.2 (0.5, 3.2) | 0.6 (0.1, 5.7)/ NE | 1.7 (0.7, 4.0)/ 1.7 (0.7, 4.1) | 1.1 (0.4, 3.4)/ 1.2 (0.4, 3.4) |
| 9. Pyloric stenosis | 1.2 (0.9, 1.7)/ 1.1 (0.7, 1.5) | 0.5 (0.2, 1.1)/ 0.8 (0.3, 1.7) | 1.1 (0.8, 1.6)/ 1.0 (0.7, 1.5) | 0.9 (0.6, 1.4)/ 0.9 (0.6, 1.4) |
| 10. Limb reduction | 0.6 (0.2, 1.8)/ 0.6 (0.2, 1.7) | 1.3 (0.4, 4.2)/ 1.8 (0.5, 6.4) | 1.3 (0.7, 2.6)/ 1.3 (0.6, 2.5) | 1.5 (0.7, 3.2)/ 1.2 (0.5, 2.8) |
| 11. Gastroschisis | 0.3 (0.1, 1.3)/ 0.3 (0.1, 1.1) | 0.3 (0.0, 2.2)/ 0.5 (0.1, 4.3) | 0.9 (0.3, 2.2)/ 0.8 (0.3, 2.1) | 1.2 (0.5, 2.8)/ 1.1 (0.5, 2.6) |
| 12. Hypospadias | 0.8 (0.6, 1.1)/ 0.8 (0.6, 1.1) | 1.0 (0.6, 1.5)/ 1.0 (0.7, 1.6) | 0.9 (0.7, 1.2)/ 0.9 (0.7, 1.2) | 0.8 (0.6, 1.1)/ 0.8 (0.6, 1.1) |

*p<0.05; NE - Not estimated.

Table S8. Adjusted PRs and CIs at the block group level excluding individuals living in public distribution areas. PRs were adjusted for maternal age, race, and education status.

| Defect | As | Cd | Mn | Pb |
| --- | --- | --- | --- | --- |
| 1. Spina bifida | 0.7 (0.2, 3.3) | NE | 2.2 (0.8, 5.7) | 2.1 (0.8, 5.5) |
| 2. Anotia/ microtia | NE | NE | NE | NE |
| 3. Conotruncals | 2.3 (1.0, 5.1)* | 1.9 (0.2, 15.1) | 2.8 (1.2, 6.4)* | 0.9 (0.3, 2.7) |
| 4. AVSD/ ECD | 1.2 (0.3, 5.4) | NE | 1.1 (0.2, 5.1) | NE |
| 5. HLHS | 1.6 (0.4, 5.7) | NE | 0.6 (0.1, 4.4) | 1.4 (0.4, 4.8) |
| 6. Cleft palate | 0.6 (0.1, 2.7) | NE | 0.6 (0.2, 2.1) | 0.9 (0.4, 2.4) |
| 7. Cleft lip ± CP | 1.0 (0.4, 2.4) | 2.0 (0.4, 9.3) | 0.6 (0.2, 1.8) | 0.9 (0.4, 2.4) |
| 8. EA/ TEF | 2.0 (0.5, 7.5) | NE | 1.4 (0.4, 5.2) | 1.0 (0.2, 4.4) |
| 9. Pyloric stenosis | 1.0 (0.6, 1.8) | 1.5 (0.5, 5.0) | 1.0 (0.6, 1.7) | 1.1 (0.6, 1.9) |
| 10. Limb reduction | 0.7 (0.2, 3.1) | 2.8 (0.3, 26.4) | 1.0 (0.3, 2.8) | 1.6 (0.5, 4.9) |
| 11. Gastroschisis | 0.5 (0.1, 2.1) | NE | 1.0 (0.3, 3.0) | 1.0 (0.3, 2.8) |
| 12. Hypospadias | 0.7 (0.5, 1.2) | 1.6 (0.8, 3.4) | 0.8 (0.5, 1.2) | 0.8 (0.5, 1.2) |

*p<0.05; NE - Not estimated.
